# Dynamics of anti-malarial antibodies in non-immune patients during and after a first and unique *Plasmodium falciparum* malaria episode

**DOI:** 10.1186/s12936-020-03300-x

**Published:** 2020-06-26

**Authors:** Zeno Bisoffi, Marco Bertoldi, Ronaldo Silva, Giulia Bertoli, Tamara Ursini, Stefania Marocco, Chiara Piubelli, Elena Pomari, Dora Buonfrate, Federico Gobbi

**Affiliations:** 1 Department of Infectious–Tropical Diseases and Microbiology, IRCCS Sacro Cuore Don Calabria Hospital, Negrar, Verona, Italy; 2 Department of Diagnostics and Public Health, University of Verona, Verona, Italy; 3 Infectious Diseases Department, University Hospital of Verona, Piazzale L.A. Scuro, Verona, Italy

**Keywords:** Malaria, *Plasmodium falciparum*, Imported, Non-immune, Antibodies, IFAT

## Abstract

**Background:**

Malaria is a major travel medicine issue. Retrospective confirmation of a malaria episode diagnosed in an endemic area can have relevant implications in transfusional medicine in Europe, where blood donors are excluded from donation on the basis of positive malaria serology. However, there is scarce evidence on the dynamics of anti-malarial antibodies after a first malaria episode in non-immune individuals. The first aim of this study was to describe the dynamics of anti-malarial antibodies in a first malaria episode in non-immune travellers. Secondary objectives were to assess the sensitivity of serology for a retrospective diagnosis in non-immune travellers diagnosed while abroad and to discuss the implications in transfusional medicine.

**Methods:**

Retrospective analysis of the results of an indirect fluorescence antibody test (IFAT) for malaria available for patients with a first malaria episode by *Plasmodium falciparum* and admitted at the IRCCS Sacro Cuore Don Calabria hospital in a 14-year period. The antibody titres were collected at baseline and during further follow up visits. Epidemiological, demographic and laboratory test results (including full blood count and malaria parasite density) were anonymously recorded in a study specific electronic Case Report Form created with OpenClinica software. Statistical analysis was performed with SAS software version 9.4.

**Results:**

Thirty-six patients were included. Among them, all but two were Europeans (one African and one American). Median length of fever before diagnosis was 2 days (IQR 1–3). Thirty-five patients had seroconversion between day 1 and day 4 from admission, and the titre showed a sharply rising titre, often to a very high level in a few days. Only a single patient remained negative in the first 5 days from admission, after which he was no more tested. Six patients were followed up for at least 2 months, and they all showed a decline in IFAT titre, tending to seroreversion (confirmed in one patient with the longest follow up, almost 4 years).

**Conclusions:**

Serology demonstrated reliable for retrospective diagnosis in non-immune travellers. The decline in the anti-malarial titre might be included in the screening algorithms of blood donors, but further studies are needed.

## Background

Malaria is the most important disease caused by protozoa in tropical and subtropical regions with an estimated burden of about 220 million cases in 2017 and about 430,000 deaths worldwide. The vast majority of cases is caused by *Plasmodium falciparum* [1]. Malaria is also a major topic in travel medicine and should be considered in all febrile patients after return from endemic areas. About 6200 imported malaria cases are reported in Europe each year [2], and in addition many travellers report a history of malaria during their stay abroad.

In endemic countries, malaria microscopy is often inaccurate or simply unavailable, and malaria remains a clinical and, therefore, presumptive diagnosis in many cases, although in recent years immune chromatographic, rapid diagnostic tests, much easier to perform and to read, not requiring a laboratory, have gradually replaced microscopy across most African endemic countries, often allowing a prompt diagnosis and management of malaria [3, 4].

Malaria serology is of no value in diagnosing acute malaria and may be still negative during a first, acute malaria episode. However, the presence of antibodies is witness of a previous malaria episode, and in non-endemic countries, particularly in Europe, serological testing of blood donors is recommended to all people having visited malaria-endemic countries. Positive donors are excluded from donation until negativization. However, recent research has questioned this approach to donor screening [5, 6].

Antibody detection can also be useful for the retrospective differential diagnosis of fever in non-immune travellers presenting after a journey to endemic areas and who report an episode of fever that was diagnosed as malaria [7, 8]. In the experience of this as well as of other centres dealing with travel medicine, many diagnoses of malaria (typically *P. falciparum* malaria) reported by returning travellers are unreliable, either because clinically based without testing, or because the quality of local diagnosis is often poor outside the few reference centres. If it would possible to show that: a) anti-malarial (anti-trophozoite, *P. falciparum*) antibody tests are invariably positive after an acute *P. falciparum* malaria episode, and b) positivity persists for at least some weeks in absence of further exposure, then the test could be retrospectively used to confirm or exclude *P. falciparum* malaria in a non-immune, recently returned traveller. However, little is known of the dynamic of anti-malarial antibodies after a first malaria episode in non-immune patients. The main purpose of this study was to retrospectively retrieve all available data on *P. falciparum* malaria antibody tests (IFAT, Bio-Mérieux) carried out on non-immune patients presenting with a first malaria episode, in order to describe the dynamic of anti-malarial antibodies. The underlying hypothesis is that, in all non-immune subjects with a first *P. falciparum* malaria episode, a detectable antibody titre invariably appears within the first few days after the onset of fever and remains detectable for at least some weeks. If this is so, then the test would be useful for the retrospective diagnosis (or exclusion) of malaria in non-immune travellers reporting a recent treatment for malaria before returning home. Moreover, serology is currently the method of choice for the screening of blood donors. Understanding the duration of the persistence of a positive titre after a single malaria episode, as well as the dynamics of the antibody titre evolution with time, may help to better inform the current policy.

The primary objective of this study was to describe the evolution of anti-malarial antibody titre during and after a first malaria episode in non-immune patients. Secondary objectives were:

a) To assess the sensitivity of this test for a retrospective diagnosis (or exclusion) of malaria in travellers diagnosed with *P. falciparum* malaria while abroad.

b) To discuss the implications of the study results on the policy of anti-malarial antibody testing as a screening tool in transfusional medicine.

Primary endpoint: results of all available anti-malarial antibody tests (IFAT BioMérieux) performed over time on patients responding to the inclusion criteria, since the first determination during the acute clinical malaria.

## Methods

Medical records of patients admitted with *P. falciparum* malaria to the Department of Infectious – Tropical Diseases and Microbiology (DITM), IRCCS Sacro Cuore - Don Calabria Hospital, Negrar, Verona, Italy, in a 14-year period between January 2004 and December 2017, were retrospectively analysed in order to identify non-immune patients presenting with a first malaria episode caused by *P. falciparum*. If anti-malarial serology was performed at least once during hospitalization, the patient was included in the study and the essential demographic, clinical and laboratory data were recorded in an anonymous database. For each recruited subject, any subsequent determinations of anti-malarial antibody titre (during admission and at any follow-up visit) was also recorded, with no time limits. History of any subsequent malaria episode as reported at follow-up visits was also recorded. The dynamic of the evolution of the antibody titre over time was analysed.

### Inclusion criteria

Adult patients with *P. falciparum* malaria on presentation, and with no previous malaria history, for which the anti-malarial (anti - *P. falciparum* blood stages) antibody titre (IFAT BioMérieux) was assessed at least once during hospitalization, between January 2004 and December 2017. Patients had to be resident in non-endemic countries for at least 10 years prior to the acute malaria episode.

### Exclusion criteria

Records with incomplete epidemiological/demographic data or clinical history. Lack of written consent to donate the required biological samples (serum) for the study purpose.

### Data management and analysis

Results of other laboratory tests performed on the same group during the acute clinical malaria (malaria parasite density, haemoglobin, WBC count, PLT count) were also recorded and so were the main demographic, clinical and epidemiological characteristics of the study population. No formal calculation of the sample size was performed, as [PLEASE NOTE THAT MALARIA JOURNAL DOES NOT USE FIRST PERSON FORMAT; ADJUST ALL SENTENCES ACCORDINGLY]ALL the records responding to the inclusion criteria in the period considered were included. Expected number was of about 40 subjects.

### Indirect Fluorescence Antibody Test (IFAT)

IFAT assay (Falciparum-Spot IF, Biomérieux Italia S.p.A.-Firenze) was performed according to the manufacturer’s instructions. Briefly, the test enables the detection of *Plasmodium*-specific (anti-asexual blood forms) total antibodies (no distinction is made between IgM and IgG) and samples are considered negative when IFAT titre = 0. In strict terms, titres express serial dilutions, where 20 (strictly speaking: 1/20) is the first dilution considered, therefore “0” simply means “negative, or no fluorescence, at the first dilution of 1/20. For clarity in the paper, titres are expressed as whole numbers, where each whole number corresponds to the denominator of the respective dilution. According with the manufacturers’ instructions, weak positivity (titre 20 to 40) is considered to be due to either a) a past infection by *P. falciparum*; b) a past or active infection by another plasmodial species (due to cross reaction with falciparum antigens). Titres ≥ 80 indicate a (past or active) *P. falciparum* malaria infection. A positive (titre 160) and a negative control are included in each session. In order to estimate its accuracy, the test was carried out on 36 blood donors with no relevant travel history and the result was negative (0 titre) for all. The test was also performed on 84 African subjects recently (up to one year before testing) arrived in Italy from endemic areas, with no acute nor recent malaria symptoms and all tested positive with an antibody titre varying between 160 and 10,120, save for two subjects with a titre of 80.

### Data management and analysis

Data were collected in a standardized electronic Case Report Forms (CRF) using OpenClinica software© (OpenClinica LLC and collaborators, Waltham, MA, USA, www.OpenClinica.com.) In the database the patients’ data were anonymized and identified by a study univocal code. Statistical analysis was performed with SAS software version 9.4. All collected data were summarized using descriptive statistics. Estimated parameters were reported with their respective 95% CI. Both, statistical methods and plots were used to assess the data.

The following main analyses were performed.

The anti-malarial antibody titre recorded from the first determination during the acute clinical malaria until the last follow-up visit was first visually assessed using plots (as the points in time were not the same for all the study subjects). In the plot, for each patient, the set of observations over time would give rise to a curve (or profile) allowing to compare the dynamic of anti-malarial antibodies across time and subjects.

At each follow-up visit, sensitivity of serology was calculated using the patients *P. falciparum* malaria known diagnosis on presentation. For subjects with a minimal follow-up of one month, the sensitivity (after a recent malaria) was calculated as the proportion: positive subjects at ≥ 1 month/all subjects.

### Ethical clearance

The study protocol was approved by the Ethical Committee (Comitato Etico per la Sperimentazione Clinica delle province di Verona e Rovigo), Protocol Number 47963.

## Results

### Study population

**Fig. 1:**
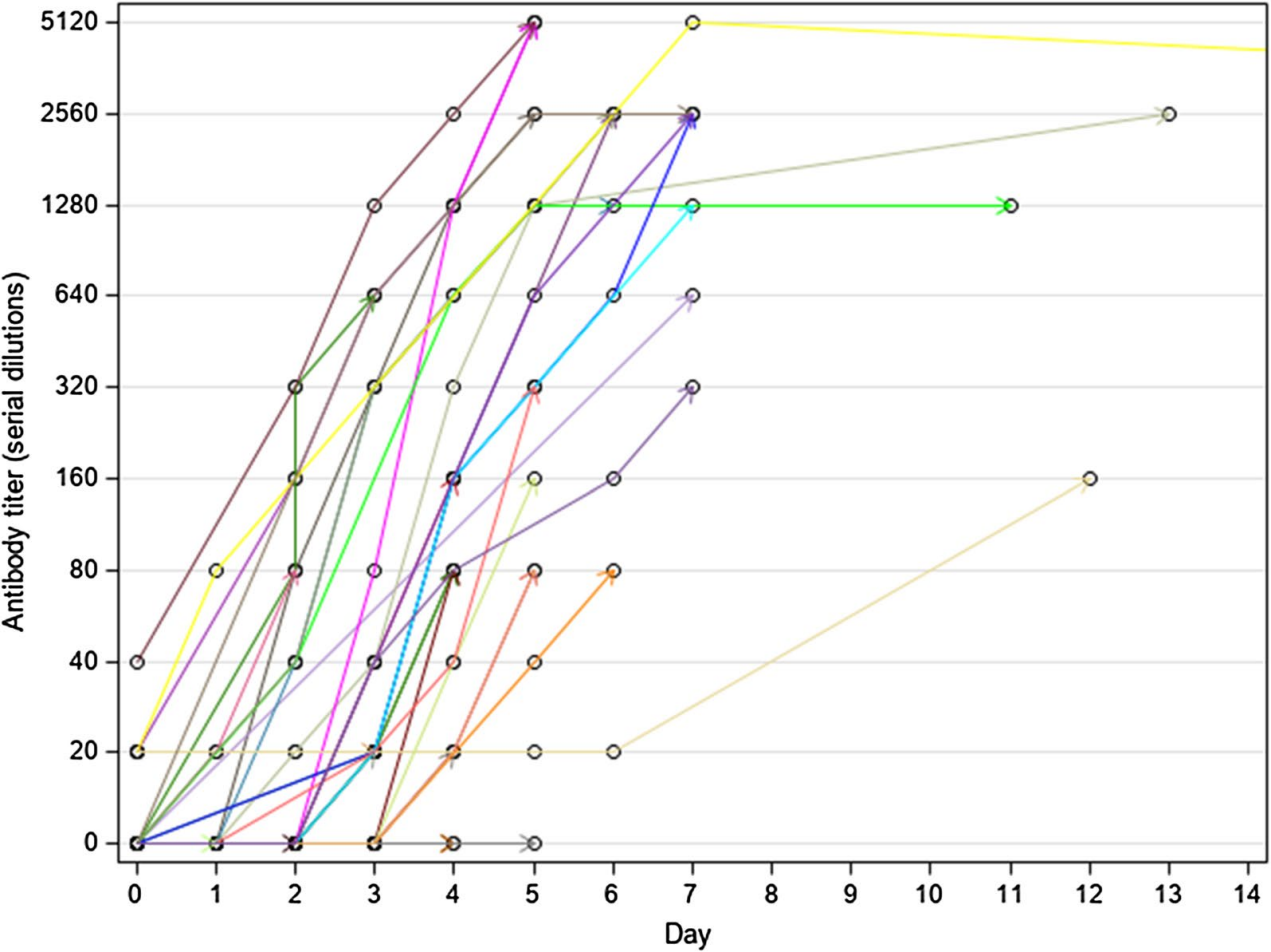
Evolution of antibody titre during hospitalization (all 36 patients, including one who was still in hospital after 2 weeks). Dots represent the time a sample was tested. Last tested sample is represented by an arrow and coincides with the last day of hospitalization

Thirty-six subjects were included. The main demographic, clinical characteristics and laboratory findings of the included patients are summarized in Table 1. There was a predominance of males. Almost all patients were European, one was African (from Nigeria) and another was American (USA). The Nigerian patient had been staying in Italy for 12 years before travelling to his country of origin (as VFR) in June 2014, when he caught malaria. The median age was 50 years. Five cases (13.9%) were classified as severe malaria. All patients were discharged without any sequelae, but one who was transferred to another hospital for dialysis and eventually was also discharged without complications. The laboratory data (Table 1) were compatible with *P. falciparum* malaria in non-immune subjects. The dynamics of anti-malarial antibody titre during hospitalization is summarized in Fig. 1.

**Table 1:** Main demographic, clinical and laboratory data

	Median (IQR) or number (%)
Demographical data	
Sex	
Female	10 (27.78%)
Male	26 (72.22%)
Age, years	50 (31–57)
Origin	
Africa	1 (2.78%)
Europe	34 (94.44%)
USA	1 (2.78%)
Clinical data	
Fever (°C) at admission	38.2 (37.05–39)
Severe malaria	5 (13.89%)
Outcome	
Discharged without complications	35 (97.22%)
Transferred to other hospitals	1 (2.78%)
Febrile days before diagnosis	2 (1–3)
Laboratory data	
Parasitaemia at diagnosis (N/µL)	18,760 (1020–172,992)
Max Parasitaemia during admission (N/µL)	18,760 (1243–215,550)
Hb at diagnosis (g/L)	14.4 (13.1–15.6)
Minimal Hb during admission (g/L)	12.6 (11.5–13.4)
Platelet count (Plt) at diagnosis (N/µL)	102,000 (68,000–146,000)
Minimal Plt during admission (N/µL)	51,000 (41,000–71,000)
White Blood Cells (N/µL)	4640 (3850–5740)

**Fig. 2:**
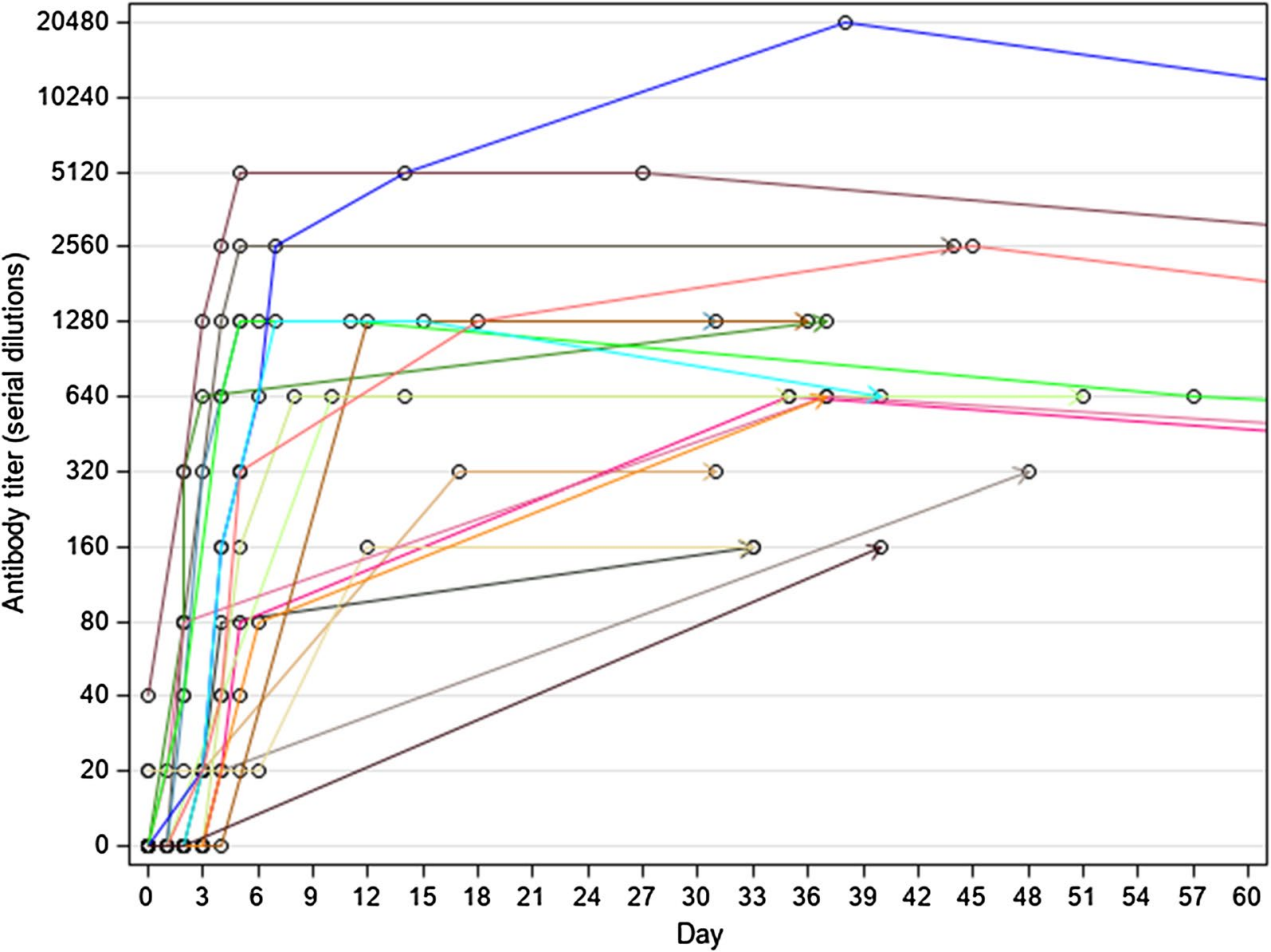
Evolution of antibody titre after discharge, until 2 months after admission (19 patients). Dots represent the time a sample was tested. Last tested sample is represented by an arrow

Briefly, 31 patients (86.1%) were still negative at the beginning of the acute malaria episode when all had already a detectable parasitaemia (varying between 14 and 1,120,000 trophozoites/µL) (Fig. 1). Only 5 patients (15, 79%) had already a detectable titre, 4 at the lower positive dilution (20) and 1 at 40. One of them was the patient from Nigeria (see above), the other four were Italians who had all a past travel history to malaria-endemic countries, although none of them reported a previous malaria episode. In all cases but one (that had a last antibody titration carried out at the fifth day after admission, with no follow-up), the antibody test became positive (or started to increase in titre if already positive) at Day 1 to Day 6 of observation, to subsequently show a sharply rising titre, often to a very high level (up to 20,480 in a few days). The only patient who remained negative during admission was an Italian traveller who was admitted on September 6^th^, 2013 with fever after a short journey to Cameroon. He had a positive RDT for *P. falciparum* and a positive Quantitative Buffy Coat (QBC), but the thick and thin films were both negative, indicating an exceedingly low parasite density. Unfortunately, it was not possible to follow up the patient after he was discharged. Nineteen patients had the last antibody titration after discharge (Fig. 2). For most of them (13) the follow-up ended within 2 months after hospitalization. All had a high antibody titre (ranging from 160 to 2560) at their last observation (30 to 51 days after admission).

**Fig. 3:**
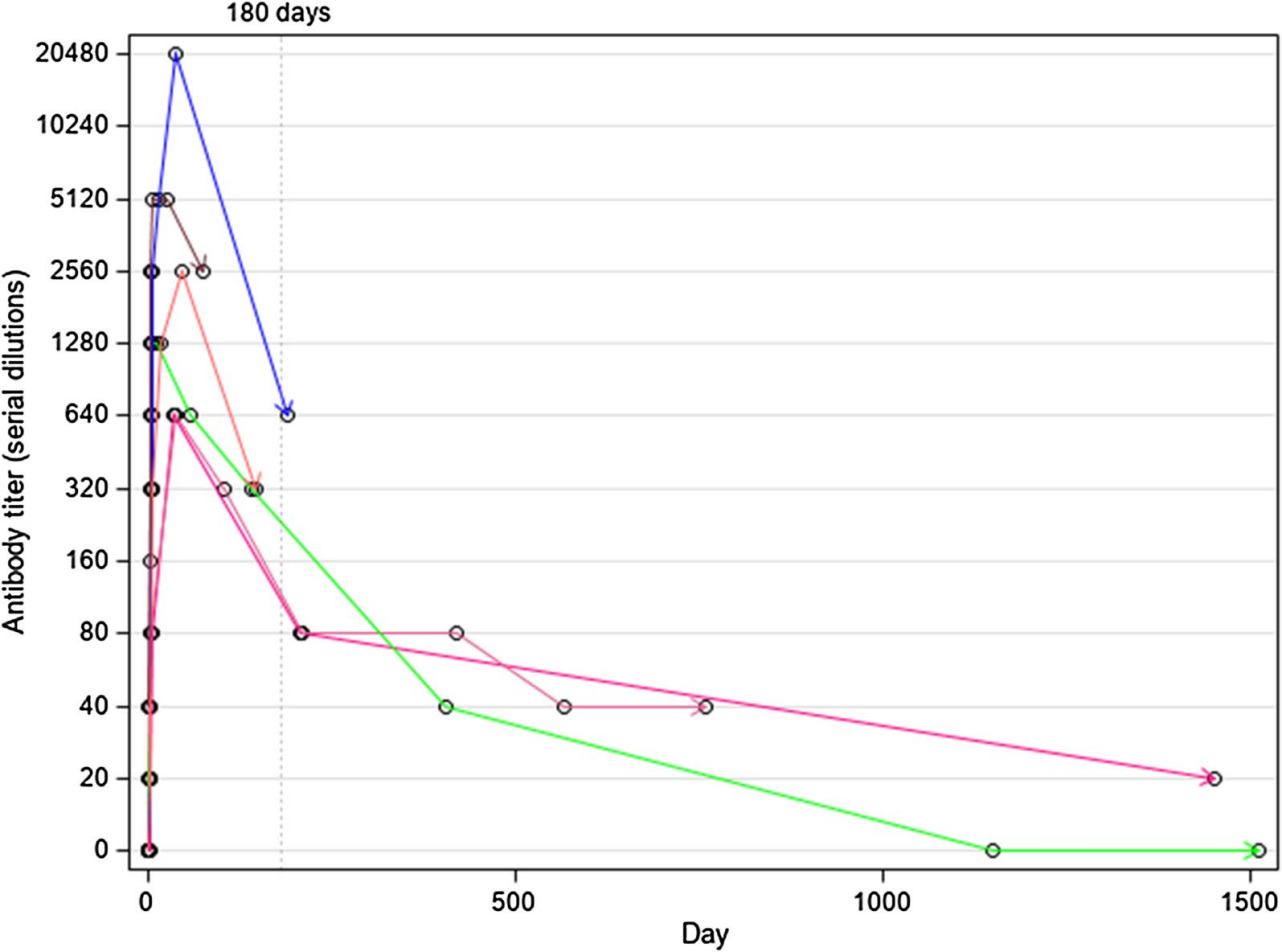
Evolution of antibody titre for the 6 patients who had a long-term follow-up. Dots represent the time a sample was tested. Last tested sample is represented by an arrow

For six subjects, it was possible to follow the antibody titre for more than 2 months (range 3 months–4 years) (Fig. 3). For all the six subjects the highest antibody titre was reached within the first two months, then declined tending to negativization. The latter was observed after about 3 years in the subject with the longest observation and confirmed at the last follow-up of 4 years, while another subject with almost as long a follow-up was still positive after almost 4 years but at the lowest titre of 1/20. The absence of any further exposure was confirmed by all patients until the last follow-up date.

The test sensitivity for the retrospective diagnosis of a recent malaria resulted 100% (19/19) for patients who had a follow-up until at least 1 month (Fig. 2).

As further exploratory analyses, correlations were searched between some key variables. A moderate positive correlation was found between the parasite density and the number of febrile days before diagnosis (Sperman Correlation Coefficient = 0.43, p = 0.01), while a weak positive correlation was found between the parasite density and the maximum antibody titre attained (Sperman C C = 0.38, p = 0.03). On the contrary, a strong negative correlation was found between the parasite density and the days to antibody positivization (Sperman C C = 0.61, p = 0.0001).

The full data set of the antibody titre over time for all study subjects is resumed in Additional file 1: Table S1. The detailed clinical and laboratory data of all patients are reported in Additional file 1: Table S1.

## Discussion

This study analysed the evolution of the anti-malarial (*P. falciparum* anti-trophozoites) antibody titre in non-immune patients suffering from their first *P. falciparum* malaria episode, which was confirmed by a negative antibody titre in almost all of them. While there is a paucity of papers describing the natural evolution of anti-malarial antibodies after a natural infection, relevant research has been done on experimental infection, often in the context of studies aimed at malaria vaccine development [9].

Recently, Boyle et al. interestingly compared the dynamics of anti-merozoite IgM and IgG following experimentally induced as well as natural malaria infection [10]. The latter, differently from this study population, was mainly composed of children and adults with repeated malaria exposure. Among other interesting findings, they discovered that IgM had a long persistence in both groups, similar to that of IgG, and that IgM in particular were likely to play a protective role by blocking merozoite invasion of erythrocytes in a complement-dependent manner. They also studied a small cohort of 10 Australian returning travellers with a (self-reported) first malaria episode, showing again persistence of IgM antibodies in all of them up to 6 months after the infection and no difference in the decline of the latter, compared with IgG, up to 300 days after infection [10].

The scope of this work was of course different. This was a non-experimental, observational study, using a commercially available anti-*P. falciparum* IFAT, principally aimed at finding a potential diagnostic (retrospective) role in returning travellers on one side, and at discussing the role of serology as a screening tool in transfusional medicine on the other. The antibodies detected by the test are total antibodies with no distinction between classes, and there are no clues in this study to discuss their protective role, that was not among the study objectives. In particular, it is not possible to state that finding a positive IFAT titre, say, at 1 year or two after the first (and unique) malaria episode would confer a protection in case of re-exposure, although it is logical to suppose that this may be partially so, in comparison with a subject with a negative titre.

To the authors’ knowledge this is the first such study published. Virtually all patients became positive during the acute malaria episode, and the positivity, after a sharp increase in titre, persisted for at least 1 month in the 19 patients who had a follow-up after discharge. Thus, a negative test result would virtually rule out a previous *P. falciparum* malaria in a returning traveller with a recent (i.e. 1 month or less) diagnosis while travelling, while a positive test would retrospectively confirm malaria. Five patients had already a detectable antibody titre at their first observation, four at the lowest titre of 20, and one at titre 40. One of them was a (VFR) patient from Africa (Nigeria) who reported no previous malaria history, although a past exposure might have escaped recollection. Unfortunately, the last available antibody titre for this patient (at the very high value of 5120) was recorded during hospitalization and no follow-up was available. Although it has been shown that immigrants, even if with a malaria history, tend to lose their semi-immunity after a long stay abroad [11, 12], the dynamic of the antibody response in this group, after a new malaria episode, would be probably different from that in completely naïve patients.

One patient remained negative until the last observation before discharge (5 days after admission). This was a patient with an exceedingly low parasite density, and given the strong negative correlation between the parasite density and the days to positivization, had the patient had a sufficient observation time a positive antibody titre would probably eventually have appeared. This cannot be stated with certainty, though, as he was unfortunately lost to follow-up after discharge. As for the five patients who had already a positive (albeit very weak) antibody titre at their first determination, a possible explanation is a previous, unreported malaria episode, or alternatively this may represent an early positivization (at least for two of them who had both a very high parasite density on admission).

In all patients with a sufficiently long follow-up, the antibody titre reached a peak in about a couple of months and then started declining, with no case of rebound, tending to negativization. This could open interesting perspectives in transfusional medicine. Current rules tend to exclude a donor who has a positive serology, irrespective of the titre. However, a declining titre most probably indicates that malaria infection is no longer present and might be taken as sufficient to re-admit a subject to donation, best if in combination with a sensitive molecular test, and without waiting for a complete negativization that would take a comparatively long time. It is reasonable to assume that chronic carriers of a very low or undetectable parasitaemia (who might represent a theoretical risk of transmission through blood donation) would maintain a high antibody titre. This is typically observed in chronic malaria infection [13]. Subjects with a persistently high antibody titre would be automatically excluded from blood donation, moreover they might benefit from an effective anti-malarial treatment, even in case of failure to detect circulating *Plasmodium* parasites, antigen or DNA [13]. Of course, a change of the current policy would need more evidence to be suggested, but a periodical serologic testing might be suggested especially for donors with particularly requested blood groups, also considering that serologic tests are available at a reasonable cost.

### Study limitations

The sample size, that is a convenience sample based on data availability and is constrained by the retrospective design of the study, might not be sufficient to adequately describe the antibody dynamic over time. However, this study provides a first indication of the trend of anti-malarial antibodies after a first, successfully treated malaria episode in non-immune subjects. The antibody tests were executed with a method (IFAT–BioMérieux) that is no longer available (unfortunately the manufacturer has ceased production). It would be interesting to validate the results with other, commercially available (ELISA) tests with the serum specimens from the same patients that are still available in the hospital biobank.

## Conclusion

Anti-malarial antibodies against *P. falciparum* blood stages invariably appear during the acute attack and rapidly increase in titre. The test is reliable for the retrospective diagnosis or exclusion of malaria in non-immune travellers. In transfusional medicine, specifically targeted, ad-hoc studies are needed to inform a possible new approach to malaria screening in donors, where, in combination with molecular diagnostics, a declining antibody titre may be considered for inclusion in the screening algorithms.

## Supplementary information

**Additional file 1: Table S1.** Main characteristics of the study population: country of origin, length of fever, laboratory findings.

## Data Availability

The datasets generated and/or analysed during the current study will be available in the Zenodo repository upon acceptance of the article.

## Abbreviations

IFAT: indirect fluorescence antibody test
DITM: Department of Infectious – Tropical Diseases and Microbiology
CRF: Case Report Forms
Plt: platelets
VFR: visiting friends and relatives
QBC: Quantitative Buffy Coat
