# Supplementary material for: Dynamics of anti-malarial antibodies in non-immune patients during and after a first and unique Plasmodium falciparum malaria episode
Source: Malar J. 2020 Jun 26;19:228. doi: 10.1186/s12936-020-03300-x (PMC7316638; doi:10.1186/s12936-020-03300-x)
Supplement: Supplementary file 1 — Additional file 1: Table S1. Main characteristics of the study population: country of origin, length of fever, laboratory findings. [file 12936_2020_3300_MOESM1_ESM.docx]

| **Patient ID** | **Origin** | **Country of exposure** | **Ab at admission** | **Ab at 1st positivity** | **Ab max** | **Fever at adimission** | **Febrile days before diagnosis** | **Parasitemia at diagnosis**  **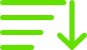** | **Day of positivity after hospitalization** | **Outcome** |
| --- | --- | --- | --- | --- | --- | --- | --- | --- | --- | --- |
| 408 | Italy | KENYA | 0 | 640 | 640 | Yes | 4 | 1120000 | 10 | 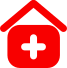 |
| A5 | Italy | COST AVORI | 0 | 640 | 2560 | No | 3 | 493000 | 3 | 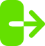 |
| A8 | Italy | CIAD | 0 | 40 | 2560 | Yes | 3 | 474300 | 3 | 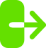 |
| A4 | Italy | missing | 20 | 80 | 5120 | missing | missing | 360000 | 1 | 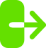 |
| 28 | Italy | SIERRA LEONE | 0 | 20 | 2560 | Yes | 4 | 324360 | 2 | 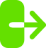 |
| 382 | Italy | UGANDA | 0 | 80 | 1280 | Yes | 4 | 312910 | 2 | 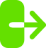 |
| 416 | Italy | BENIN | 0 | 40 | 1280 | Yes | 1 | 287910 | 2 | 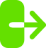 |
| A9 | Italy | MALI | 0 | 40 | 320 | Yes | 2 | 254250 | 3 | 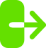 |
| 423 | Italy | KENYA | 0 | 20 | 20480 | Yes | 1 | 215550 | 3 | 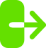 |
| 109 | Africa | NIGERIA | 20 | 160 | 5120 | Yes | 3 | 172992 | 2 | 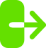 |
| 142 | Italy | CAMERUN | 0 | 80 | 2560 | Yes | 4 | 161920 | 2 | 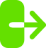 |
| A10 | USA | R.CENTR | 0 | 20 | 2560 | Yes | 2 | 136000 | 1 | 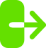 |
| 108 | Italy | SUD AFRICA | 0 | 40 | 160 | Yes | 5 | 123000 | 3 | 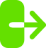 |
| 165 | Italy | TANZANIA | 0 | 40 | 1280 | Yes | 4 | 111035 | 2 | 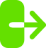 |
| 415 | Italy | TOGO | 0 | 20 | 640 | Yes | 2 | 110250 | 1 | 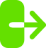 |
| 150 | Italy | TOGO | 0 | 20 | 80 | Yes | 1 | 83664 | 3 | 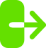 |
| 125 | Italy | CAMERUN | 0 | 20 | 20 | Yes | 2 | 25313 | 3 | 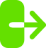 |
| 383 | Italy | SIERRA LEONE | 0 | 80 | 5120 | Yes | 2 | 18760 | 3 | 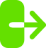 |
| 357 | Italy | BURKINA FASO | 0 | 80 | 80 | Yes | 0 | 17890 | 4 | 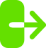 |
| 453 | Italy | BENIN | 0 | 20 | 1280 | Yes | 2 | 13728 | 3 | 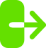 |
| A6 | Italy | NIGERIA | 0 | 160 | 160 | Yes | 0 | 13533 | 40 | 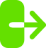 |
| 251 | Italy | TANZANIA | 0 | 20 | 2560 | Yes | 2 | 12822 | 3 | 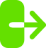 |
| A7 | Italy | ETIOPIA | 40 | 320 | 5120 | Yes | 2 | 7900 | 2 | 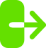 |
| 140 | Italy | SIERRA LEONE | 0 | 20 | 160 | Yes | 2 | 3448.55 | 3 | 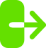 |
| A2 | Italy | TANZANIA | 0 | 20 | 2560 | Yes | 1 | 3140 | 3 | 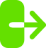 |
| 139 | Italy | TOGO | 0 | 20 | 320 | Yes | 2 | 2190 | 4 | 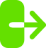 |
| A1 | Italy | MALI | 0 | 40 | 640 | Yes | 1 | 1243 | 5 | 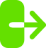 |
| 63 | Italy | UGANDA | 0 | 40 | 640 | Yes | 0 | 1020 | 4 | 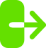 |
| 426 | Italy | MALI | 0 | 1280 | 1280 | Yes | 1 | 666 | 12 | 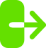 |
| A3 | Italy | TOGO | 0 | 20 | 640 | Yes | 2 | 428 | 4 | 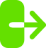 |
| 451 | Italy | CONGO | 0 | 160 | 160 | Yes | 2 | 86 | 11 | 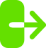 |
| 107 | Italy | SIERRA LEONE | 20 | 320 | 320 | Yes | 1 | 46 | 17 | 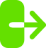 |
| 155 | Italy | SIERRA LEONE | 0 | 640 | 640 | Yes | 4 | 14 | 7 | 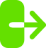 |
| 254 | Italy | REP CENTRO AFRICANA | 0 | 20 | 640 | Yes | 2 | 2 | 4 | 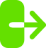 |
| 121 | Italy | CAMERUN | 0 | 0 | 0 | Yes | 1 | missing | 4 | 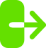 |
| 414 | Italy | MALI | 20 | 160 | 160 | Yes | 1 | missing | 12 |  |


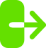
= Discharged without complications
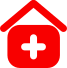
 = Transferred to other hospital
